# Bilateral Sacrospinous Colposuspension with Sling for Advanced Pelvic Organ Prolapse: Anatomical and Functional Outcomes in a 235-Patient Cohort

**DOI:** 10.3390/jcm15114295

**Published:** 2026-06-02

**Authors:** Irene Sánchez-Urbaneja, Elisa M. López-Herrero-Pérez, Francisco Rivas-Ruiz, Raquel Romero-Pérez, María José Núñez-Matas, Ana Astorga-Zambrana, Laura M. Palomar-Sánchez

**Affiliations:** 1 Department of Obstetrics and Gynecology, Virgen de la Victoria University Hospital, 29010 Málaga, Spain; elopezherrerop2@gmail.com (E.M.L.-H.-P.);; 2 Department of Gynecology and Obstetrics, Faculty of Medicine, University of Málaga, 29010 Málaga, Spain; 3 IBIMA Plataforma Bionand, Instituto de Investigación Biomédica de Málaga, 29590 Málaga, Spain; francisco.rivas.ruiz.sspa@juntadeandalucia.es; 4 Research and Innovation Unit, Hospital Universitario Costa del Sol, 29603 Marbella, Spain

**Keywords:** pelvic organ prolapse, bilateral sacrospinous fixation, vaginal surgery, sling, apical prolapse repair

## Abstract

**Background:** Pelvic organ prolapse (POP) is a prevalent condition that often requires surgical correction of apical support. Vaginal approaches that restore anatomy while minimizing synthetic material are of increasing clinical interest. Bilateral sacrospinous colposuspension with sling has been proposed as a minimally invasive technique; however, evidence from large clinical cohorts remains limited. **Objectives:** This study aimed to evaluate the anatomical, functional, and safety outcomes of this procedure in women with symptomatic advanced POP. **Methods:** This retrospective single-center cohort study included 235 consecutive women who underwent bilateral sacrospinous colposuspension with sling for symptomatic POP between 2018 and 2024. The primary outcomes were anatomical success (Baden stage ≤ II) and functional success (absence of vaginal bulge symptoms). Secondary outcomes included urinary, bowel, and sexual function, patient satisfaction, and postoperative complications classified according to the Clavien–Dindo system. **Results:** At a median follow-up of 20 months, anatomical success was achieved in 87.1% of patients and functional success in 93.6%. Significant improvements were observed in POP-Q points Ba and C (*p* < 0.001). Among symptomatic patients, stress urinary incontinence improved in 66%, urgency in 63%, and constipation in 71%. Overall morbidity was low (5.5%), with most complications classified as Clavien–Dindo grade I–II. Mesh extrusion occurred in 2.1% of cases, and reintervention was required in 2.1%. Functional recurrence was observed in 6.4% of patients, with 26% requiring surgical reintervention. Patient satisfaction was high (median score: 9/10). **Conclusions:** These findings support bilateral sacrospinous colposuspension with sling as a safe and effective vaginal approach for symptomatic advanced POP; however, the retrospective design and absence of a control group should be considered when interpreting the results.

## 1. Introduction

Pelvic organ prolapse is defined as the descent of one or more pelvic organs into the vaginal canal and is associated with substantial impairment in quality of life due to symptoms of pelvic pressure, vaginal bulge, and urinary, bowel, and sexual dysfunction. The true prevalence of POP remains difficult to establish, as reported rates vary widely depending on whether symptomatic criteria or anatomical findings are used, ranging from approximately 3–6% in symptomatic women to nearly 50% when based on physical examination findings [1,2].

In advanced POP, most patients undergoing surgical repair present with multicompartment defects. Clinically significant apical prolapse frequently coexists with advanced anterior and/or posterior compartment prolapse, and surgical correction should therefore address these defects simultaneously to improve the durability of repair [3]. The main surgical objective is to restore normal vaginal anatomy while preserving or improving pelvic floor function. Current evidence recognizes adequate suspension of the vaginal apex as a key element in pelvic floor reconstructive surgery, since apical support contributes substantially to anatomical stability and may reduce the risk of medium- and long-term recurrence. A multicompartment reconstructive approach has been associated with improved anatomical outcomes and lower recurrence rates compared with isolated compartment repair, particularly in women with large anterior wall defects who may benefit from concomitant colporrhaphy [4].

Beyond mechanical failure of pelvic support structures, advanced POP has also been associated with increased vulnerability of vaginal connective tissue due to alterations in the extracellular matrix. These changes, influenced by aging, hypoestrogenism, and chronic mechanical stress, may contribute to disease progression and recurrence after native tissue repair, particularly in postmenopausal women or those with levator ani defects [5,6]. Consequently, surgical techniques that provide additional support while minimizing the amount of implanted synthetic material may be particularly advantageous [7,8].

Furthermore, the stability of prolapse repair depends not only on restoring apical support but also on re-establishing a physiological vaginal morphology [9]. Imaging-based models have demonstrated that distortion of the vaginal axis and position is associated with prolapse recurrence, supporting surgical strategies aimed at maintaining a centered vaginal axis and a symmetrical configuration of the upper vagina while preserving vaginal length and function [10,11].

Different procedures for apical suspension have been described using either vaginal or abdominal approaches. However, the vaginal route remains the most commonly used approach in many reconstructive series and is frequently considered a first-line option in prolapse surgery because it allows concomitant correction of associated compartment defects with limited surgical invasiveness [1,2]. Sacrospinous fixation, introduced in the 1950s, represented an important advance in vaginal apical suspension and can be effectively combined with anterior and/or posterior colporrhaphy.

Classically, sacrospinous fixation has been performed unilaterally under direct vision through posterior colpotomy and pararectal dissection to access the sacrospinous ligament. However, this approach has been associated with potential risks of rectal injury, neurovascular complications, and postoperative pain, possibly related to extensive dissection. More recently, suture-capturing devices have allowed fixation to the sacrospinous ligament with minimal dissection, potentially reducing these procedure-related risks [12].

Sacrospinous fixation remains a widely used technique for apical prolapse repair; however, recurrence reported with unilateral approaches has prompted interest in bilateral suspension techniques. Unilateral fixation may posteriorly deviate the vaginal axis and has been associated particularly with recurrence in the anterior compartment [12,13,14,15]. In this context, Kieback [16] first described the use of an ultralight macroporous sling for apical suspension designed to act as a scaffold and distribute tension more symmetrically. Subsequent studies have reported encouraging anatomical and functional outcomes with a favorable safety profile [17,18].

However, clinical evidence evaluating this technique in large patient cohorts remains limited. Therefore, the aim of this study was to evaluate the anatomical, functional, and safety outcomes of bilateral sacrospinous colposuspension with sling in women with pelvic organ prolapse.

## 2. Materials and Methods

### 2.1. Study Design and Population

A retrospective monocentric cohort study was conducted including women who underwent bilateral sacrospinous colposuspension with sling for symptomatic pelvic organ prolapse at our institution between 2018 and 2024. Eligible patients were aged ≥18 years and presented symptomatic pelvic organ prolapse requiring apical support correction. Patients were identified from the institutional surgical database, and clinical information was obtained from electronic medical records, operative reports, and postoperative follow-up visits. The study population therefore reflects consecutive clinical practice in a tertiary pelvic floor unit.

Inclusion criteria were symptomatic primary or recurrent pelvic organ prolapse with apical involvement (stage II or greater) and failure or intolerance of conservative treatment. Exclusion criteria included previous pelvic floor surgery with synthetic mesh, known allergy to prosthetic material, or contraindication for surgery. Baseline variables collected included age, parity, body mass index, comorbidities including hypertension, diabetes mellitus, chronic respiratory disease, fibromyalgia, anxiety or depression, smoking status, sexual activity, previous pelvic surgery, prolapse stage according to both the Baden–Walker classification and the POP-Q system, presence of levator ani muscle injury, and type of concomitant procedure performed.

The study was approved by the local institutional review board [approval number: number 146-12-2024], and the requirement for informed consent was waived due to the retrospective design of the study.

### 2.2. Preoperative Evaluation

All patients underwent a standardized preoperative assessment including detailed medical history, focused pelvic floor examination, and evaluation of urinary, bowel, and sexual symptoms. Prolapse severity was assessed using the Pelvic Organ Prolapse Quantification (POP-Q) system and the Baden classification. Pelvic examination was performed in the lithotomy position, including maximal Valsalva maneuver, and each vaginal compartment was evaluated separately in order to identify the dominant defect and determine the need for concomitant anterior or posterior repair.

When indicated, transvaginal or transperineal ultrasound was performed to evaluate pelvic floor structures and associated pelvic pathology. The indication was agreed upon through Shared Decision-Making (SDM) [19]. During preoperative counselling, patients were informed about available conservative and surgical alternatives, including pessary use, native tissue repair, abdominal or laparoscopic apical suspension procedures, and the potential risks and benefits of mesh-assisted apical suspension. The final surgical indication was based on prolapse severity, symptom burden, anatomical findings, patient preference, and suitability for surgery.

### 2.3. Surgical Technique

All procedures were performed by surgeons experienced in pelvic floor reconstructive surgery. Patients were placed in the lithotomy position under regional or general anesthesia, and a Foley catheter was inserted. Standard antiseptic preparation and sterile draping were performed before vaginal access. The surgical field was exposed using vaginal retractors, and careful attention was paid to identifying the vaginal apex or cervix, the pararectal spaces, and the anatomical relationship with the ischial spines and sacrospinous ligaments.

The choice between the anterior and posterior approach to the sacrospinous ligament was determined according to the anatomical distribution of the prolapse. In patients without a relevant posterior compartment defect, an anterior approach was preferred to avoid unnecessary dissection of the posterior vaginal wall. Conversely, in patients with a posterior compartment defect and a marked anterior compartment component, a posterior approach was selected because it allowed a wider anterior colporrhaphy to be performed when required, which may be technically more limited through an anterior approach.

After infiltration of the vaginal mucosa with a solution containing a local anesthetic and vasoconstrictor, the corresponding vaginal incision was made, followed by blunt bilateral dissection toward the sacrospinous ligaments. Dissection was limited to the minimum required to expose the fixation area and allow safe passage of the sutures, with the aim of reducing bleeding, rectal injury, and postoperative pain. Hemostasis was checked throughout the procedure.

Bilateral sacrospinous colposuspension was then performed using the BSC Mesh (A.M.I. Agency for Medical Innovations GmbH, Feldkirch, Austria). This mesh is composed of a special ultralight, isoelastic, monofilament polypropylene with a macroporous structure. Its weight is approximately 21 g/m^2^ and its porosity reaches 93%, characteristics intended to facilitate tissue ingrowth while minimizing the foreign-body reaction. The macroporous design also reduces the amount of material in direct contact with the vaginal wall, with an estimated contact surface of approximately 3 cm^2^. The mesh has a U-shaped morphology and is passed from one sacrospinous ligament to the contralateral ligament, embracing the vaginal vault or cervix in a tension-free sling-like configuration. This bilateral support is based on the integral theory and pelvic support architecture, and is designed to restore apical support while maintaining the vaginal axis in a more physiological position. Fixation to the sacrospinous ligaments is performed using the I-Stitch device.

**Figure 1:**
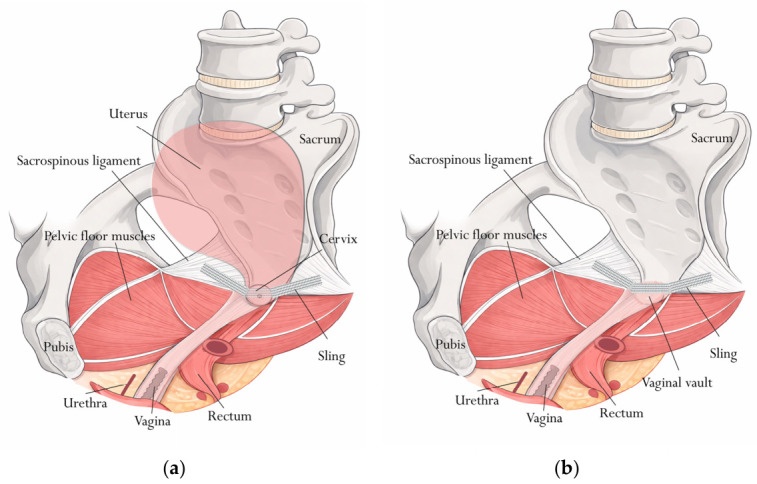
Schematic illustration of bilateral sacrospinous procedures with sling. (**a**) Schematic illustration of hysteropexy with sling, showing uterine preservation and bilateral fixation to the sacrospinous ligaments. (**b**) Schematic illustration of colpopexy with sling in hysterectomized patients, demonstrating vaginal vault suspension with bilateral sacrospinous fixation. The figure highlights sling placement and restoration of apical support.

After bilateral identification of the sacrospinous ligaments, sutures were placed in each ligament using the dedicated i-Stitch suturing device. Sutures were placed under digital guidance after palpation of the ischial spine and sacrospinous ligament, avoiding excessive lateral placement in order to reduce the risk of neurovascular injury. The sutures were attached to the lateral arms of the mesh, whereas the central portion was secured either to the cervix in women undergoing uterine preservation or to the vaginal apex in those with prior hysterectomy (Figure 1). The mesh was placed in a tension-free fashion to restore apical support while maintaining a centralized vaginal axis. Before closure, the absence of excessive tension, folding, or vaginal wall exposure of the mesh was confirmed.

Concomitant procedures, including anterior and/or posterior colporrhaphy, perineoplasty, or mid-urethral sling placement, were performed when clinically indicated. The indication for concomitant mid-urethral sling placement was based on the presence of symptomatic stress urinary incontinence, confirmed by clinical examination and 3D transperineal ultrasound. All eligible patients were counselled that the anti-incontinence procedure could be performed either as a separate staged procedure or during the same surgical session. Therefore, a mid-urethral sling was performed only in patients who fulfilled the diagnostic criteria for stress urinary incontinence and elected to undergo concomitant surgical treatment. The vaginal incision was then closed with absorbable sutures.

### 2.4. Postoperative Care and Follow-Up

All patients received perioperative antibiotic prophylaxis and thromboprophylaxis according to institutional protocols. A Foley catheter and vaginal packing were maintained for 12–24 h postoperatively. Patients were discharged once adequate spontaneous voiding and clinical stability were confirmed. Postoperative recommendations included avoidance of heavy lifting, intense physical activity, and vaginal intercourse during the early healing period, according to institutional postoperative instructions. Patients were advised to seek medical assessment in case of fever, abnormal bleeding, pelvic pain, voiding difficulty, or symptoms suggestive of infection or mesh exposure.

Postoperative follow-up was performed according to routine clinical practice. Patients were generally evaluated at 2–3 months and at 12 months after surgery, with annual follow-up recommended thereafter for up to 3 years. During follow-up, patients underwent pelvic examination to assess anatomical outcomes, including POP-Q assessment when available, and were systematically questioned regarding urinary, bowel, and sexual symptoms. Patient-reported sensation of vaginal bulge and overall satisfaction with the surgical procedure were also recorded. When recurrence was suspected, anatomical findings were documented by compartment, and the presence or absence of recurrent bulge symptoms was recorded to distinguish objective from symptomatic recurrence.

### 2.5. Outcome Measures

The primary outcomes were anatomical and functional success. Anatomical success was defined as pelvic organ prolapse stage ≤ II according to the Baden classification at follow-up examination. Functional success was defined as the absence of patient-reported vaginal bulge symptoms.

Secondary outcomes included the safety profile of the procedure and changes in pelvic floor symptoms. Intraoperative complications recorded included bleeding and bladder injury. Postoperative complications included chronic pelvic pain, dyspareunia, mesh extrusion or erosion, and surgical reintervention. Chronic pelvic pain was defined as pelvic pain persisting for more than three months after surgery. All complications were classified according to the Clavien–Dindo classification. Mesh exposure was defined as visible or palpable prosthetic material through the vaginal epithelium during follow-up examination. Surgical failure was considered when recurrent prolapse required further surgical treatment or when symptomatic recurrence was documented during follow-up.

Changes in pelvic floor symptoms, including urinary, bowel, and sexual function, were assessed during follow-up visits through systematic clinical questioning. Patient satisfaction with the surgical procedure was evaluated using a numerical satisfaction scale ranging from 0 to 10, where higher scores indicated greater satisfaction.

### 2.6. Statistical Analysis

Data analysis was performed using IBM SPSS Statistics for Windows, Version 28.0. Descriptive statistics were used to summarize patient characteristics and surgical outcomes. Continuous variables were expressed as mean ± standard deviation or median with interquartile range, as appropriate. The distribution of continuous variables was assessed before selecting parametric or non-parametric tests. Categorical variables were summarized as absolute frequencies and percentages.

Preoperative and postoperative POP-Q measurements were compared using the Wilcoxon signed-rank test. Differences in age according to categorical variables were assessed using non-parametric tests: the Mann–Whitney U test for dichotomous variables and the Kruskal–Wallis test for variables with more than two categories. Statistical significance was defined as *p* < 0.05. Missing data were not imputed, and analyses were performed using available cases for each variable.

## 3. Results

### 3.1. Patient Characteristics

A total of 235 women underwent bilateral sacrospinous colposuspension with sling for symptomatic pelvic organ prolapse between 2018 and 2024. The median follow-up period was 20 months.

Baseline characteristics of the study population are summarized in Table 1. The mean age at the time of surgery was 63.7 years, and the mean body mass index (BMI) was 27.2 kg/m^2^. Hypertension was present in 40.9% of patients and diabetes mellitus in 13.6%. The median parity was two deliveries, with a predominance of vaginal births. Previous pelvic surgery had been performed in 20% of patients, most commonly abdominal hysterectomy (11.5%).

At baseline, the median Baden stage was III, with 94.9% of patients presenting advanced prolapse (stage III–IV). In patients with available imaging, levator ani muscle defects were identified in more than 75% of cases.

### 3.2. Surgical Characteristics

Surgical characteristics are presented in Table 2. The surgical approach was predominantly posterior (68.1%), and most procedures were combined with concomitant anterior (91.9%) and posterior colporrhaphy (94.9%). A mid-urethral sling was placed in 8.9% of patients.

General anesthesia was required in less than 1% of cases. The median operative time was 90 min, and hospital stay was short, with a median of 1 day. Intraoperative complications were uncommon (3.8%), with bladder injury being the most frequent event.

### 3.3. Anatomical and Functional Outcomes

Surgical outcomes are summarized in Table 3. Anatomical success, defined as Baden stage ≤ II, was achieved in 87.1% of patients, while functional success, defined as the absence of vaginal bulge symptoms, was reported in 93.6%. Significant postoperative improvements were observed in POP-Q points Ba and C. Median Ba improved from 3 cm preoperatively (IQR 2.5 to 4) to −1 cm postoperatively (IQR −2 to 0), while median C improved from 1 cm preoperatively (IQR −2 to 3) to −5 cm postoperatively (IQR −6 to −4). Both comparisons reached statistical significance (*p* < 0.001). Total vaginal length was largely preserved, although a small statistically significant postoperative reduction was observed, from 9.01 ± 1.17 cm preoperatively to 8.27 ± 1.01 cm postoperatively, with a mean paired difference of 0.74 cm (95% CI 0.49 to 0.99; *p* < 0.001). Preoperative and postoperative POP-Q measurements are presented in Table 4.

Functional outcomes were favorable, with improvement reported in 66% of patients with stress urinary incontinence, 63% with urgency symptoms, and 17% with constipation. Patient satisfaction was high, with a median score of 9 (0–10 scale) and more than 70% of patients reporting a satisfaction score ≥9.

**Figure 2:**
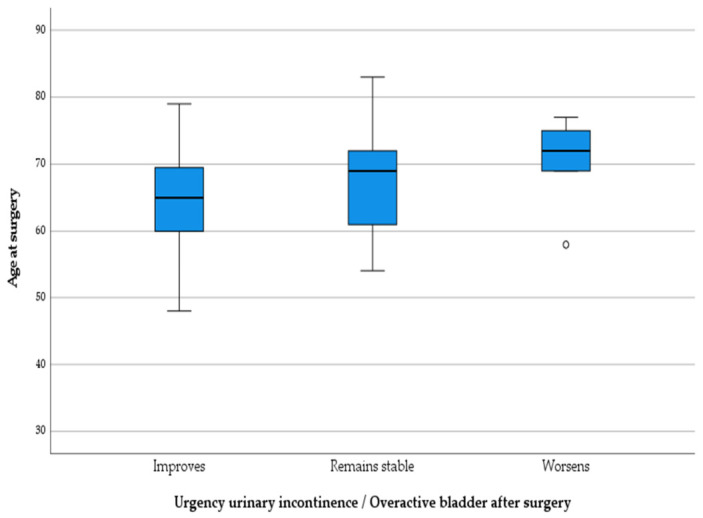
Boxplot showing patient age according to clinical outcome (improved, unchanged, or worsened). The horizontal line within each box represents the median, and the small circle indicates an outlier.

Additionally, analysis of the relationships between variables demonstrated that increasing age was significantly associated with poorer postoperative outcomes in urgency urinary incontinence, with a progressive gradient observed across the improvement, stable, and worsening groups (*p* = 0.026), as shown in Figure 2. Patients in the stable/worsening groups were older than those who improved (median 69 vs. 65 years; *p* = 0.013).

**Figure 3:**
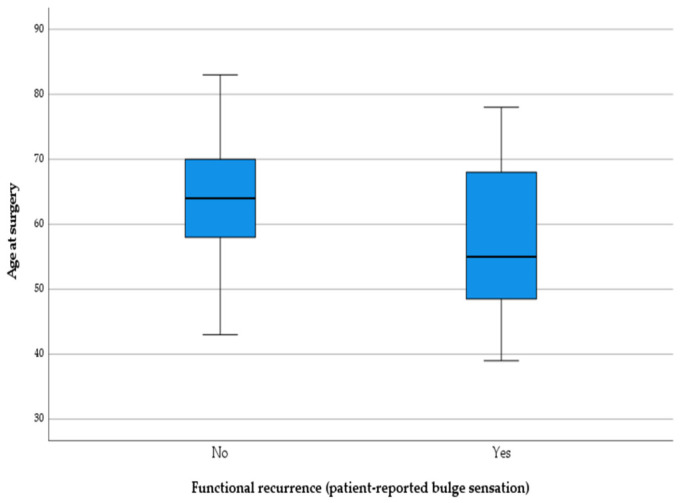
Boxplot showing patient age according to the presence or absence of functional recurrence. The horizontal line within each box represents the median.

Patients with functional recurrence were significantly older than those without functional recurrence (*p* = 0.02), as shown in Figure 3.

### 3.4. Complications

Intraoperative complications occurred in 3.8% of cases and included bladder injury (*n* = 7) and intraoperative bleeding (*n* = 2). Postoperative morbidity was low, with 13 complications recorded (5.6%). Most events were mild (Clavien–Dindo grade I), including dyspareunia (1.7%) and chronic pelvic pain (0.85%), both managed with standard analgesic treatment. Mesh extrusion or erosion occurred in 2.1% of patients; most cases were treated conservatively (Clavien–Dindo II), while a minority (0.85%) required surgical excision under anesthesia (Clavien–Dindo IIIb). The overall surgical reintervention rate was 2.1%, including procedures for functional recurrence and mesh extrusion. Postoperative complications are summarized in Table 5.

## 4. Discussion

In this study, bilateral sacrospinous colposuspension with sling demonstrated high anatomical and functional success rates with a low complication profile in women with advanced pelvic organ prolapse. Anatomical success (Baden ≤ II) was achieved in 87.1% of patients, while functional success, defined as the absence of vaginal bulge symptoms, was reported in 93.6%. In addition to restoring apical support, the procedure was associated with improvement in urinary and bowel symptoms and high patient satisfaction. These findings support the effectiveness of this technique as a minimally invasive vaginal approach for the management of advanced and multicompartment pelvic organ prolapse. Importantly, our cohort represents one of the largest reported series evaluating bilateral sacrospinous colposuspension with sling.

Our results are consistent with previous reports evaluating sacrospinous colposuspension using ultralight macroporous slings. Kieback [16] first described this approach as a minimally invasive strategy to provide apical support while distributing tension symmetrically through bilateral fixation. Subsequent clinical series have reported encouraging outcomes. Chene et al. described anatomical success rates exceeding 90% with very low mesh-related complications [17], while Hosni et al. similarly reported high success rates and significant improvements in quality-of-life measures with minimal erosion rates [18]. The results of the present study are in line with these findings, demonstrating high anatomical and functional success together with a favorable safety profile in a large cohort of patients.

The favorable outcomes observed in our series may also be explained by the biomechanical principles underlying bilateral sacrospinous suspension. Unilateral sacrospinous fixation has been associated with posterior deviation of the vaginal axis and increased tension on the anterior compartment, which may contribute to anterior compartment recurrence. In contrast, bilateral suspension with a sling may provide a more symmetrical distribution of forces and help maintain a centered vaginal axis. Restoration of a physiological vaginal orientation has been shown to be an important determinant of durable prolapse repair. By anchoring the sling bilaterally to the sacrospinous ligaments and securing it to the cervix or vaginal apex, this technique may act as a scaffold that reinforces native support structures while preserving vaginal length and function.

The safety profile observed in this study is also noteworthy. The overall rate of postoperative complications was low, with most events classified as Clavien–Dindo grade I and managed conservatively. Mesh-related complications were infrequent, with an extrusion rate of 2.1%, and only a small proportion of cases required surgical excision. These findings are consistent with previous reports evaluating ultralight macroporous slings for apical suspension, which have demonstrated low rates of mesh-related morbidity compared with earlier generations of transvaginal mesh systems. The limited amount of implanted material and the tension-free configuration of the sling may contribute to reducing the inflammatory response and minimizing the risk of mesh exposure. In addition, the low reintervention rate observed in our series further supports the safety and durability of this approach.

This study has several strengths. First, it includes a large cohort of patients undergoing bilateral sacrospinous colposuspension with sling, representing one of the largest series reported for this technique. Second, the analysis incorporated both anatomical and functional outcomes, including patient-reported symptoms and satisfaction, providing a comprehensive assessment of surgical effectiveness. Additionally, the evaluation of urinary, bowel, and sexual symptoms allows a broader understanding of the functional impact of prolapse repair beyond anatomical correction alone. However, some limitations should also be acknowledged. The retrospective design may introduce inherent sources of bias, and the absence of a control group prevents direct comparison with other surgical techniques for apical prolapse repair. Furthermore, postoperative follow-up was performed according to routine clinical practice rather than a strictly predefined study protocol, which may have introduced variability in follow-up timing and outcome assessment. Functional outcomes were assessed through systematic clinical questioning rather than validated pelvic floor symptom questionnaires, which may also introduce reporting bias. Although the follow-up period was sufficient to evaluate early and mid-term outcomes, longer prospective comparative studies with standardized follow-up protocols and validated symptom questionnaires are needed to better assess the long-term durability and functional impact of this approach.

From a clinical perspective, these findings may have practical implications for the surgical management of advanced pelvic organ prolapse, particularly in patients requiring apical support with concomitant correction of anterior or posterior compartment defects. Bilateral sacrospinous colposuspension with sling may represent an intermediate approach between native tissue repair and more extensive mesh-based reconstructive procedures, as it aims to provide symmetrical apical support while limiting the amount of implanted material. This technique may be especially relevant in patients in whom restoration of the vaginal axis, preservation of vaginal length, and maintenance of vaginal function are surgical priorities. In addition, the vaginal route allows simultaneous treatment of multicompartment prolapse without the need for an abdominal approach, which may be advantageous in selected patients with surgical risk factors or preference for less invasive procedures.

Future research should prioritize prospective studies and, ideally, randomized controlled trials comparing bilateral sacrospinous colposuspension with sling to other established techniques for apical prolapse repair, given the limited direct comparative evidence currently available. These comparisons should include conventional unilateral sacrospinous fixation, native tissue vaginal repair, and abdominal or laparoscopic sacrocolpopexy in order to better define the relative benefits and limitations of each approach.

Extended follow-up is warranted to better assess long-term durability, particularly regarding recurrence rates reported after other vaginal procedures. Longer follow-up is also required to evaluate late recurrence, mesh-related complications, reoperation rates, sexual function, and patient-reported outcomes over time. The integration of advanced imaging techniques and biomechanical analyses may further elucidate the impact of vaginal axis restoration on surgical outcomes. In particular, imaging-based studies may help clarify whether restoration of a centered vaginal axis translates into improved long-term anatomical and functional results.

The systematic inclusion of patient-reported outcomes, including quality of life, sexual function, and shared decision-making, is essential to ensure a patient-centered evaluation. Finally, multicenter studies and real-world data will be key to enhancing external validity and confirming the reproducibility of these findings across different clinical settings.

## 5. Conclusions

Bilateral sacrospinous colposuspension with sling appears to be an effective and safe surgical option for the treatment of advanced pelvic organ prolapse. In this large cohort, the technique achieved high anatomical and functional success rates with a low complication profile and high patient satisfaction. These findings support its role as a minimally invasive vaginal approach capable of restoring apical support while preserving vaginal function. Further prospective studies with longer follow-up are warranted to confirm the long-term durability of this technique.

## Figures and Tables

**Table 1:** Baseline characteristics of the study population.

Variable	Value
Age (years), mean ± SD	63.7 ± 8.78
BMI ^1^ (kg/m^2^), mean ± SD	27.2 ± 4.23
Hypertension	96 (40.9%)
Diabetes mellitus	32 (13.6%)
Chronic respiratory disease	16 (6.8%)
Fibromyalgia	11 (4.7%)
Anxiety or depression	47 (19.6%)
Current smokers	33 (14.0%)
Sexually active	127 (54.0%)
Parity (*n*), median (IQR ^2^)	2 (2–3)
Previous pelvic surgery	47 (20.0%)
Previous abdominal hysterectomy	27 (11.5%)
Baden III–IV	223 (94.9%)
Levator ani muscle defect	181 (77.0%)

^1^ BMI: Body Mass Index. ^2^ IQR: interquartile range (p25–p75).

**Table 2:** Surgical characteristics of the study population.

Variable	Value
Anterior approach	75 (31.9%)
Posterior approach	160 (68.1%)
Concomitant anterior colporrhaphy	219 (91.9%)
Posterior colporrhaphy and/or perineoplasty	223 (94.9%)
Cervical amputation	8 (3.4%)
Dual sacrospinous fixation	13 (5.5%)
Concomitant mid-urethral sling	21 (8.9%)
General anesthesia	2 (0.9%)
Operative time (min), median (IQR ^1^)	90 (80–116)
Hospital stay (days), median (IQR)	1 (1–1)

^1^ IQR: interquartile range (p25–p75).

**Table 3:** Anatomical and functional outcomes.

Variable	Value
Anatomical success (Baden ≤ II)	87.1%
Functional success (absence of vaginal bulge)	93.6%
Improvement in stress urinary incontinence	66%
Improvement in urgency symptoms	63%
Improvement in constipation	17%
Satisfaction score (0–10), median (IQR)	9 (8–10)
Patients with satisfaction ≥ 9	>70%

**Table 4:** Preoperative and postoperative POP-Q measurements of Ba, C, and total vaginal length.

POP-Q ^1^ Point	Preoperative Value (cm)	Postoperative Value (cm)	*p*-Value
Ba, median (IQR)	3 (2.5 to 4)	−1 (−2 to 0)	<0.001
C, median (IQR)	1 (−2 to 3)	−5 (−6 to −4)	<0.001
TVL ^2^, mean ± SD	9.01 ± 1.17	8.27 ± 1.01	<0.001

Values are expressed as median (interquartile range) for Ba and C, and as mean ± standard deviation for TVL. Preoperative and postoperative Ba, C and TVL values were compared using the Wilcoxon signed-rank test. ^1^ POP-Q: Pelvic Organ Prolapse Quantification. ^2^ TVL: total vaginal length.

**Table 5:** Postoperative complications classified according to the Clavien–Dindo classification.

Clavien–Dindo Grade	Postoperative Complication	*n* (%)
I	Dyspareunia	4 (1.7%)
	Chronic pelvic pain (>3 months)	2 (0.85%)
II	Mesh exposure/erosion managed conservatively	3 (1.3%)
IIIb	Mesh extrusion requiring surgical excision	2 (0.85%)
	Reintervention for functional recurrence	4 (1.7%)

## Data Availability

The data supporting the findings of this study are not publicly available due to privacy and ethical restrictions but are available from the corresponding author upon reasonable request.

## Abbreviations

The following abbreviations are used in this manuscript:

POP	Pelvic organ prolapse
POP-Q	Pelvic Organ Prolapse Quantification system
SMD	Shared Decision-Making
BMI	Body Mass Index
IQR	Interquartile range (p25–p75)
